# The Impact of Living Kidney Donor Glomerular Filtration Rate on Graft Survival

**DOI:** 10.3390/medicina61040580

**Published:** 2025-03-25

**Authors:** Umit Cakmak, Ozgur Merhametsiz, Nurettin Ay

**Affiliations:** 1 Antalya Memorial Hospital, Nephrology Clinic, 07025 Antalya, Turkey; 2 Nephrology Department, Faculty of Medicine, Yeni Yüzyıl University, Private Gaziosmanpaşa Hospital, 34245 İstanbul, Turkey; ozgurmerhametsiz@gmail.com; 3 Gazi Yasargil Training and Research Hospital, Transplantation Department, University of Health Sciences, 42130 Diyarbakir, Turkey; nurettinay1977@gmail.com

**Keywords:** kidney transplantation, graft survival, graft loss, mortality, donor

## Abstract

*Background and Objectives:* Kidney transplantation (KT) is the gold-standard treatment for end-stage renal disease (ESRD). An accurate evaluation of donor renal function is critical for living kidney transplantation. This study aimed to assess the impact of donor glomerular filtration rate (GFR) on long-term graft function and survival. *Materials and Methods:* A retrospective analysis was conducted on 229 donor–recipient pairs who underwent living kidney transplantation at our center. Data on recipient demographics, clinical characteristics, and donor GFR were collected. The outcomes of graft survival were compared based on donor and recipient characteristics. Logistic regression was used to identify the factors influencing graft loss. *Results:* Mortality was significantly higher in the graft loss group (26% vs. 5.1%, *p* = 0.001). Graft biopsy was associated with a 24.74-fold increased risk of graft loss (95% CI: 5.387–113.609, *p* < 0.001). Each 1 mL/min/1.73 m^2^ increase in donor GFR reduced graft loss risk by 0.075% (95% CI: 0.870–0.983, *p* = 0.013). Donor age, gender, and BMI showed no significant impact on graft survival. *Conclusions:* A higher donor GFR positively influenced graft survival. However, donor age, gender, and BMI did not significantly affect long-term outcomes. These findings emphasize the importance of thorough donor evaluation to optimize graft survival and recipient outcomes.

## 1. Introduction

Chronic kidney disease (CKD) represents a significant global health issue, affecting over 10% of the general population worldwide [1]. Approximately 2% of patients with CKD progress to end-stage renal disease (ESRD) [2]. Kidney transplantation (KT) is considered the gold-standard treatment for patients with ESRD, offering substantial improvements in both the quality of life and survival rates [2,3].

Assessing renal function in potential living kidney donors is critical for the success of living kidney transplantations. Compared to cadaveric kidney transplantation, living kidney transplantation offers several advantages for recipients, including improved human leukocyte antigen (HLA) compatibility, a lower incidence of delayed graft function, and better graft survival rates [4]. Conversely, the extraction of a kidney from a healthy individual involves significant risks, requiring a comprehensive evaluation of potential hazards for the donor. The evaluation process for potential living kidney donors includes various tests, with the assessment of kidney function being the most critical element. To ensure that the donor retains adequate renal function for the remainder of their life after kidney transplantation and that the recipient has sufficient graft function post-transplantation, it is essential to assess the donor’s renal function using appropriate methods prior to transplantation [5]. According to the Kidney Disease Improving Global Outcomes (KDIGO) clinical practice guideline, the initial assessment is conducted by estimating the glomerular filtration rate (GFR) from serum creatinine [6]. According to the aforementioned guidelines, a GFR of ≥90 mL/min/1.73 m^2^ is considered acceptable for a donor, whereas a GFR of <60 mL/min/1.73 m^2^ is deemed unacceptable. The British Transplantation Society (BTS) guidelines utilize an assessment method that closely resembles the KDIGO approach, although the recommended threshold levels differ based on gender and age [7].

Nonetheless, a coordinated effort must be made to accurately assess the kidney function of the potential donor. Furthermore, it is essential to ensure that the donor will not develop CKD or other illnesses following kidney transplantation. It is equally important to verify that both the donor and recipient will experience long-term survival.

Although donor age, sex, and body mass index (BMI) have been investigated as potential factors influencing graft survival, existing studies report conflicting results. While some research indicates that a donor age over 60 years negatively impacts graft survival due to nephron loss and age-related renal function decline [8], other studies suggest that carefully selected older donors can achieve comparable outcomes to younger donors [9]. Similarly, while some studies suggest that grafts from male donors perform better due to larger nephron mass, others report no significant impact of donor sex on long-term graft survival [10]. The role of BMI in donor selection is also debated, with a higher BMI being linked to an increased risk of metabolic complications and nephron hypertrophy, which may affect recipient outcomes [11]. The objective of this study was to investigate the impact of donor GFR on long-term graft function and survival following living kidney transplants performed at our institution.

## 2. Materials and Methods

A retrospective analysis of the clinical data from all couples (n = 289) who underwent living kidney donor transplantation at our organ transplant center between January 2012 and December 2022 was conducted. The study was approved by the Ethics Committee of Health Sciences University, Diyarbakır Gazi Yasargil Education and Research Hospital. (date and clinical trial number: 2023-545, 13/10).

Participants in the study were patients who had been followed for a minimum of one year after kidney transplantation. Eight patients were excluded from the study due to perioperative complications, six due to primary non-functioning kidneys, 24 patients under the age of 18 years, 6 with a history of multiple organ transplantation, and 16 patients who did not attend follow-up appointments regularly. The remaining 229 pairs formed the study group.

The baseline donor GFR was estimated using the 2009 Chronic Kidney Disease Epidemiology (CKD-EPI) Collaboration equation, which is based on serum creatinine levels measured during the living donor assessment [12]. The CKD-EPI equation was chosen over the Modification of Diet in Renal Disease (MDRD) equation due to its lower bias, higher precision, and greater accuracy in estimating renal function among potential kidney donors compared to the MDRD equation [6]. A multifaceted approach was employed in the process of donor selection, encompassing a thorough evaluation of renal scintigraphy and computed tomography (CT) angiography findings. This comprehensive assessment facilitated the determination of the donor nephrectomy side. Furthermore, the confirmation of GFR involved the use of various methodologies, including 24 h urine tests, the MDRD method, and the CKD-EPI equation.

Hypertension was defined as follows: a systolic blood pressure (BP) measurement of 140 mmHg or greater, and a diastolic BP measurement of 90 mmHg or greater, as determined by at least three separate measurements; a 24 h ambulatory BP measurement of 135 mmHg or greater, recorded by a medical professional; and a documented history of hypertension or a current prescription for antihypertensive medication.

Given anatomical considerations, left-sided harvesting was the preferred approach, unless complex vascular anatomy or significant renal asymmetry was observed. The transperitoneal laparoscopic approach was employed in the majority of donors.

A comprehensive data collection process was conducted, encompassing both demographic and clinical information for all study participants. The presence of concomitant medical conditions was carefully documented. The etiologies of CKD were classified into nine categories: diabetic nephropathy (DN), hypertension (HT), glomerulonephritis (GN), polycystic kidney disease (PKD), obstructive pathologies, tubulointerstitial nephritis (TIN), hereditary conditions, amyloidosis, unknown etiologies, and other causes.

Acute rejection (AR) was defined based on biopsy criteria. Recipients were monitored until death, graft loss, or the completion of a follow-up period of at least one year. Graft survival was defined as the time until the return to dialysis, re-transplantation, or graft loss, in the event of overall patient survival. The study population was categorized into two groups: recipients and donors, with a distinction made between those who experienced graft loss and those who did not. Graft loss was defined by the occurrence of either a minimum of three consecutive months of dialysis treatment or a new kidney transplant. The occurrence of graft loss was documented along with the recording of death.

The recipient’s demographic and clinical characteristics included age, gender, BMI, primary kidney disease, history of HT, diabetes mellitus (DM), and coronary artery disease prior to kidney transplantation, blood type, preemptive status, duration of dialysis, induction immunosuppression protocol following transplantation, creatinine and GFR at discharge, creatinine and GFR at the last visit, and whether the recipient had undergone graft biopsy. The results of the graft biopsy were retrieved from the records.

A comparative analysis was conducted of donor demographic and clinical characteristics in relation to recipient graft loss. The following data were retrieved from the records: donor age, gender, BMI, type of donor operation (laparoscopic or open surgery), localization and number of kidney arteries, and pre-transplant GFR. A comprehensive evaluation was conducted to determine the correlation between the donors’ GFR at discharge and the subsequent follow-up creatinine (Cr) and GFR levels. Additionally, a logistic regression analysis was performed to identify the risk factors associated with graft loss.

The research data were entered into the computer environment using IBM SPSS Statistics version 23 (IBM Statistical Package for Social Sciences) and analyzed. Descriptive statistics for categorical variables were presented as frequencies and percentages. The chi-square test (Pearson’s chi-square) and Yates’ continuity correction were applied for categorical variables. Descriptive statistics for numerical variables were presented as the mean (±standard deviation) for normally distributed variables and median (range) for non-normally distributed variables. The normality of numerical variables was assessed using the Kolmogorov–Smirnov and Shapiro–Wilk tests. The Independent Samples *t*-test was used for normally distributed variables and the Mann–Whitney U test for non-normally distributed variables for comparing numerical variables between two independent groups. For comparisons involving more than two categorical groups, *p*-values were adjusted using the Bonferroni correction. The Spearman correlation test was used to assess the relationship between donor GFR, GFR at discharge, and GFR at the last visit of the kidney recipient. Univariate and multivariate logistic regression analyses were performed to identify independent risk factors for graft loss. Additionally, donor and recipient sex pairs were compared to assess clinical outcomes based on sex differences. For comparisons of more than two groups, a one-way ANOVA was used for normally distributed variables, and Kruskal–Wallis for non-normally distributed variables. Statistical significance was set at *p* < 0.05.

## 3. Results

The demographic and clinical characteristics of recipients in relation to graft loss are presented in Table 1. Graft biopsy was performed more frequently in the graft loss group (73.3% vs. 19.6%, *p* < 0.001). Creatinine levels and GFR at the last visit significantly differed between the groups (*p* < 0.001 for both). Tacrolimus use was similar between groups (98.1% vs. 93.3%, *p* = 0.753), while MMF was more frequently used in the graft-loss group (73.3% vs. 53.7%, *p* = 0.140) and MYF use was lower in the graft-loss group (26.7% vs. 44.9%, *p* = 0.170), though none of these differences were statistically significant. Mortality was also higher in the graft loss group (26% vs. 5.1%, *p* = 0.001) (Table 2).

As shown in Table 3, a correlation analysis was performed between the initial GFR of donors and subsequent graft function in kidney recipients, both at discharge and during the most recent visit. A moderate negative correlation was found between donor GFR and recipient creatinine at discharge (r = −0.219, *p* = 0.001). A moderate-to-strong positive correlation was observed between donor GFR and recipient GFR at discharge (r = 0.255, *p* < 0.001). Additionally, a moderate-to-strong negative correlation was identified between donor GFR and recipient creatinine at the last visit (r = −0.240, *p* < 0.001). Finally, a moderate-to-strong positive correlation was noted between donor GFR and recipient GFR at the last visit (r = 0.302, *p* < 0.001).

Logistic regression analysis was performed to identify independent risk factors associated with graft loss, as shown in Table 4. Univariate analysis was conducted using independent variables from Table 1 with *p* < 0.250, while multivariate analysis was carried out using variables with *p* < 0.100 from the univariate analysis. The results of the univariate logistic regression analysis indicated that the presence of HT in the kidney recipient prior to transplantation and graft biopsy post-transplantation were statistically significant (*p* = 0.009, *p* < 0.001, respectively). For the multivariate analysis, the following variables were included: induction therapy, pre-transplant hemodialysis (HD) in the recipient, graft biopsy, donor blood type, and donor GFR. The multivariate model was found to be statistically significant (*p* < 0.001). According to this model, the presence of HT prior to transplantation increased the risk of graft loss by 7.479-fold (95% CI: 1.937–28.882, *p* = 0.004). The performance of a graft biopsy was associated with a 24.74-fold higher risk of graft loss, although this risk was not causally related (95% CI: 5.387–113.609, *p* < 0.001). Furthermore, an increase of 1 mL/min/1.73 m^2^ in donor GFR was associated with a 0.075% reduction in the risk of graft loss (95% CI: 0.870–0.983, *p* = 0.013).

**Figure 1:**
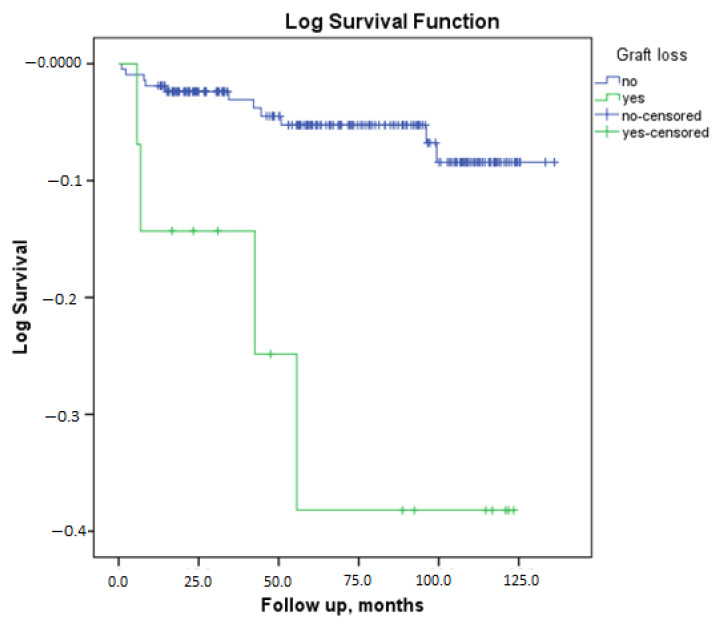
Patient survival analysis according to graft loss.

Kaplan–Meier patient survival analysis according to graft loss revealed a statistically significant difference between the groups. The patient survival duration was found to be 129.4 months (95%CI: 125.5–133.3) in the absence of graft loss and 94.2 months (95%CI: 69.9–118.5) in the presence of graft loss (*p* = 0.001) (Figure 1).

Groups according to gender matching are compared in Table 5. When the recipient was male, the number of mismatches was higher in the female donor group (3.5 vs. 3, *p* = 0.025). Furthermore, the recipient DM was higher in the female donor group (27.9% vs. 11.4%, *p* = 0.048). Conversely, when the recipient was female, male donors were found to be older (37.5 vs. 31, *p* = 0.008), the number of mismatches was higher in the male donor group (3.5 vs. 3, *p* = 0.008), recipient DM was higher in the male donor group (27.1% vs. 7.1%, *p* = 0.014), and BMI was higher in the female donor group (27.6 kg/m^2^ vs. 25.6 kg/m^2^, *p* = 0.035). A subsequent pair-wise group comparison revealed a statistically significant difference between the groups in terms of age (*p* = 0.023). This discrepancy can be attributed to the observed difference between the female donor to female recipient and male donor to female recipient groups (*p* = 0.042). Furthermore, the pairwise group comparison revealed a discrepancy between the groups in terms of mismatch (*p* = 0.004). This discrepancy was attributed to the difference between the groups from donor female to recipient female and from donor female to recipient male (*p* = 0.012).

The results of the pairwise group comparison revealed a statistically significant difference between the groups in terms of creatinine at discharge (*p* < 0.001). This discrepancy was attributed to the observed difference between the groups of donor male to recipient female and donor male to recipient male (*p* < 0.001); donor male to recipient female and donor female to recipient male (*p* < 0.001); donor female to recipient female and donor male to recipient male (*p* = 0.003); and donor female to recipient female and donor female to recipient male (*p* < 0.001). In the pairwise group comparison, a statistically significant difference was observed between the groups in terms of creatinine at the last visit (*p* < 0.001). This discrepancy was attributed to the variation between the donor male to recipient female and donor male to recipient male groups (*p* = 0.003), donor male to recipient female and donor female to recipient male groups (*p* < 0.001), donor female to recipient female and donor male to recipient female groups (*p* = 0.004), and donor female to recipient female and donor female to recipient male groups (*p* < 0.001). In the pairwise group comparison in terms of DM, a statistically significant difference was observed between the groups (*p* = 0.014). This discrepancy was attributed to the variation between the donor female to recipient male and donor female to recipient female group.

**Figure 2:**
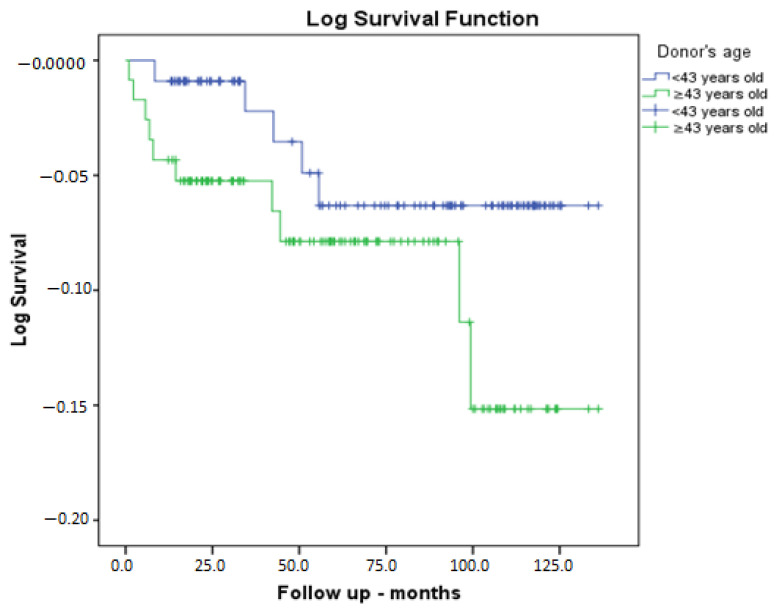
Patient survival analysis according to the median donor age of 43 years.

Donor age was divided into two groups according to median age, and accordingly, the clinical outcomes and some characteristics are given in Table 6. The results indicate that the GFR of donors in the age group under 43 years old exhibited higher levels of functioning (118 mL/min/1.73 m^2^ vs. 107 mL/min/1.73 m^2^, *p* = 0.001). Furthermore, the BMI of donors was lower in the younger age group (25.9 kg/m^2^ vs. 27.1 kg/m^2^, *p* = 0.001). Furthermore, recipient GFR at discharge was higher in the younger age group (88.3 mL/min/1.73 m^2^ vs. 79.4 mL/min/1.73 m^2^, *p* = 0.009). Furthermore, recipient creatinine levels at the final follow-up visit were found to be lower in the younger age group (1.09 mg/dL vs. 1.24 mg/dL, *p* = 0.026). Furthermore, the GFR of the recipients was found to be higher in the younger age group (75 mL/min/1.73 m^2^ vs. 65.5 mL/min/1.73 m^2^, *p* = 0.008). Furthermore, the follow-up period was found to be longer in the younger age group (78.6 months vs. 59 months, *p* = 0.029). However, there was no statistically significant difference between the two groups in terms of graft loss or mortality. The Kaplan–Meier patient survival analysis, according to the median donor age of 43 years, demonstrated no statistically significant discrepancy between the groups (*p* = 0.170) (Figure 2).

## 4. Discussion

Living kidney transplantation provides significant benefits to recipients, including improved organ quality and a shorter duration of cold ischemia. Furthermore, living kidney transplantations can be performed preemptively, reducing the duration of dialysis for patients on waiting lists. The most significant finding of this study on living kidney transplants performed at our organ transplant center is that an increase of 1 mL/min in donor GFR results in a 0.075% reduction in the risk of graft loss. In the present study, we found that other donor demographic characteristics, including age, gender, and BMI, had no significant effect on graft survival.

Numerous studies have been conducted to ascertain the correlation between donor age and recipient graft function and survival. The findings from these studies have yielded varied results, necessitating further investigation to clarify the underlying mechanisms and ensure optimal patient outcomes. A body of literature has emerged suggesting a negative correlation between donor kidney age and both graft survival outcomes and patient survival, as well as graft survival and the incidence of acute rejection. Conversely, other studies have not found a significant association between donor age and graft survival [13,14,15]. In a large-scale study by Rizzarri et al., 1762 living donor pairs were evaluated. The study found that a donor age between 56 and 65 years was not a risk factor for recipient or graft survival. However, a donor age over 65 years was associated with poorer outcomes. A subsequent analysis of recipients aged 50 years and older within the same study group revealed no differences in outcomes based on the donor’s age. However, receiving a living kidney from an older donor has been associated with adverse outcomes, including reduced patient survival and increased graft loss [13]. Aging kidneys exhibit increased interstitial fibrosis and arteriolar hyalinosis, as well as diminished nephron mass. Furthermore, older kidneys exhibit a reduced capacity for adaptive changes in response to physiological and pathological stimuli, which may further exacerbate the decline in remaining nephrons [15].

In the present study, the impact of donor age on graft function and patient survival, particularly in long-term outcomes, was examined. The majority of the living donor population was young, limiting the ability to conduct a more in-depth analysis of the age disparity between recipients and donors. This limitation, among others, hindered the ability to assess the influence of donor age on graft survival. Additionally, the complexity of isolating the impact of age in living donors from other significant variables influencing prognosis further compounded the challenges of the study.

The kidney is a sexually dimorphic organ [16]. In 1942, Dr. John Lattimer demonstrated that testosterone levels are positively correlated with inulin clearance in men and that kidney mass is testosterone-dependent, with compensatory hypertrophy increasing by 10% in both animals and humans with one kidney [17]. Given the androgen-dependent nature of renal function, it is anticipated that these functions will perform better in male recipients [18]. The study by Jacobs et al. demonstrated superior renal function in male recipients. Furthermore, the same study found that the recipients of grafts from male donors had a significantly higher graft survival rate three years after transplantation compared to recipients of grafts from female donors [19]. In a separate study, the most successful transplants were observed when male donors donated to male recipients, followed by transplants from male donors to female recipients. Conversely, the least successful transplants occurred when the donor was female and the recipient was male [20].

The present study aimed to examine the impact of donor gender on graft function and survival. The analysis revealed no statistically significant effect of donor gender on either graft function or survival. Furthermore, a subgroup analysis was performed to determine whether there was a significant difference in graft survival based on whether the donor and recipient were of the same or different gender. The results of this analysis also showed that there was no significant difference. One underlying factor contributing to this phenomenon is the observation that nephron mass from female donors, when transplanted into male recipients, results in a relatively lower renal clearance but higher eGFR on a mass basis [10]. This assertion is contradicted by the observation that male donor kidneys with a large nephron mass do not reach their full potential when transplanted into smaller female recipients. This diminished graft function may be attributed to the relative androgen deprivation inherent in the male kidney. Additional factors influencing testosterone levels in recipients include end-stage renal failure, age, comorbidities, concomitant medications, and, notably, immunosuppressive regimens [21].

Obesity has been shown to induce a series of structural, hemodynamic, and metabolic alterations in the kidneys. In obese individuals, a clinical condition known as obesity-associated glomerulopathy is observed, characterized by glomerular hypertrophy, focal segmental glomerulosclerosis, and loss of focal podocyte foot processes [22]. In adapting this model to the context of transplantation, the following hypothesis was proposed: donor BMI may influence recipient graft function. Donor kidneys with a reduced nephron mass demonstrated inferior functionality in recipients with elevated BMI, whereas those with an increased nephron mass exhibited superior functionality in recipients with a lower BMI in long-term outcomes [23]. As indicated by the existing literature, graft recipients from non-obese donors exhibited a 27% lower risk of developing delayed graft function (DGF) compared to those from obese donors [24,25,26]. A comprehensive analysis revealed no statistically significant differences in the incidence of acute rejection among renal graft recipients from donors with BMI < 30 and BMI > 30 [27].

Provenzano et al. highlighted the role of predictive and prognostic biomarkers in assessing kidney function and graft survival [28]. They emphasized that eGFR and albuminuria remain critical in evaluating long-term outcomes in chronic kidney disease and transplantation. Moreover, emerging biomarkers related to inflammation and oxidative stress could provide additional predictive power for transplant outcomes. Their findings suggest that while eGFR remains a cornerstone in risk assessment, future studies incorporating novel biomarkers may refine donor selection and post-transplant monitoring strategies

The increased probability of graft failure observed in obese donors may be attributed to obesity-related glomerular and structural damage, which renders these grafts more vulnerable to the ischemic, immune, and workload challenges of kidney transplantation. Furthermore, the elevated risk of delayed graft function in kidneys from obese donors may affect long-term outcomes. In the present study, we found that BMI had no effect on graft function and survival. An assessment of renal function is crucial in living donor evaluation. This metric serves two purposes: first, it is used for screening kidney disease, and second, it helps predict post-donation graft function and the long-term risk of kidney failure. The findings of our study indicate that for every 1 mL/min/1.73 m^2^ increase in donor GFR, there is a 0.075% reduction in the risk of graft loss. Similarly, a study by Almeida et al. demonstrated that kidney transplant recipients from living donors with high pre-transplant eGFRs (≥90 mL/min/1.73 m^2^) exhibited higher graft survival rates compared to those receiving grafts from living donors with lower eGFRs (<90 mL/min/1.73 m^2^) [29]. Norden et al. reported that a low donor GFR is associated with an increased risk of graft loss [30]. As a result, many transplant centers have established a lower acceptance threshold of 80 mL/min. However, another study found no significant difference in graft survival between kidneys harvested from living donors with an eGFR < 80 mL/min/1.73 m^2^ and those with a higher eGFR [31]. Additionally, Savoye et al. examined the impact of donor type and age on post-transplant outcomes, and found that living donor recipients aged ≥ 60 years benefited most from living donor transplantation, even when the donor was also aged ≥ 60 years [32]. Their analysis revealed that, compared to donation after brain death (DBD) grafts, living donor grafts resulted in a higher proportion of recipients achieving an estimated GFR ≥ 60 mL/min/1.73 m^2^ at one year post-transplant. These findings highlight the importance of considering donor age and function when evaluating graft suitability.

The limitations of our study include its single-center, non-randomized, retrospective design, and the lack of comparison of proteinuria follow-up in post-transplant kidney recipients with donor demographic characteristics. Furthermore, we acknowledge that the small sample size of the graft loss group (n = 15) reduces statistical power and may introduce errors when comparing subgroups. Additionally, potential residual confounders such as donor comorbidities, lifestyle factors, and unmeasured environmental influences were not accounted for in our analysis, which may have impacted the observed associations.

## 5. Conclusions

It is challenging to determine the impact of specific factors on allograft function due to the wide range of variables associated with both donors and recipients. Our study found that a higher donor GFR positively influenced graft survival. However, donor age, gender, and BMI did not have a significant long-term effect on graft survival. Further studies are needed to identify optimal donor–recipient matches and guide the development of international living donor exchange programs. These programs could facilitate matched pair exchanges, increasing opportunities and improving outcomes.

## Figures and Tables

**Table 1:** Recipient demographic characteristics and clinical characteristics.

Parameter	No Graft Loss (n = 214)	Graft Loss Is Present (n = 15)	*p*
Recipient gender, female/male (%)	87/127 (40.7%)	3/12 (20%)	0.113 ^a^
Recipient age	35 (18–69)	25 (18–63)	0.084 ^b^
Recipient BMI, kg/m^2^	22.6 (15.8–39.8)	21.3 (16.8–26.1)	0.076 ^b^
Induction			
No	11 (5.1%)	0 (0%)	0.129 ^a^
Simulect	52 (24.3%)	7 (46.7%)	
Anti-thymocyte globulin (ATG)	151 (70.6%)	8 (53.3%)	
Creatinine at discharge (mg/dL)	1.09 (0.45–5.2)	1.16 (0.65–6.58)	0.438 ^b^
GFR at discharge (mL/dk/1.73 mL^2^)	83.7 ± 24.2	82.9 ± 39.9	0.941 ^c^
Tacrolimus, yes/no (yes %)	210/4 (98.1%)	14/1 (93.3%)	0.753
Mycofenolat mofetil (MMF), yes/no (yes %)	115/99 (53.7%)	11/4 (73.3%)	0.140
Mycopheloic acid (MYF), yes/no (yes %)	96/118 (44.9%)	4/11 (26.7%)	0.170
Final creatinine	1.14 (0.54–4.1)	6.1 (1.76–12.2)	<0.001 ^b^
Final GFR	72 (14–135)	11 (5–51)	<0.001 ^b^
Greft biopsy, yes/no (%)	42/172 (19.6%)	11/4 (73.3%)	<0.001 ^a^
Biopsy result			
Normal	11 (%26.8)	0 (%0)	
Rejection	16 (%39)	7 (%63.6)	0.288 ^a^
BK virus-associated Nephropathy(BKVAN)	2 (%4.9)	1 (%9.1)	
GN	7 (%17.1)	1 (%9.1)	
Chronic allograft nephropathy(CAN)	5 (%12.2)	2 (%18.2)	
Recipient blood group			
0	61 (28.5%)	6 (40%)	
A	83 (38.8%)	3 (20%)	0.250 ^a^
B	52 (24.3%)	3 (20%)	
AB	18 (8.4%)	3 (20%)	
Time spent on dialysis, months	2 (0–240)	8 (0–120)	0.286 ^b^
Preemptive, Yes/No (%)	84/130 (539.3)	5/10 (33.3%)	0.649 ^a^
Number of HLA mismatches	3 (0–6)	3 (0–6)	0.696 ^b^
Primary kidney disease			
DN	40 (18.7%)	1 (6.7%)	
HT	14 (6.5%)	0 (0%)	
GN	19 (8.9%)	2 (13.3%)	
Obstructive pathologies	19 (8.9%)	1 (6.7%)	0.477 ^a^
PKD	3 (1.4%)	0 (0%)	
TIN	5 (2.3%)	0 (0%)	
Amyloidosis	4 (1.9%)	0 (0%)	
Hereditary	2 (0.9%)	1 (6.7%)	
Unknown etiologies	108 (50.5%)	10 (66.7%)	
Pre-tranplantation			
recipient HT, Yes/No (%)	56/158 (26.2%)	9/6 (60%)	0.005 ^a^
DM	12/2022 (5.6%)	0/15 (0%)	0.346 ^a^
Coronary artery disease(CAD)	13/201 (6.1%)	2/13 (13.3%)	0.256 ^a^
Post-transplantation			
HT, Yes/No (%)	150/64 (70.1%)	13/2 (86.7%)	0.171 ^a^
DM	47/167 (22%)	2/13 (13.3%)	0.431 ^a^
Coronary artery disease(CAD)	13/201 (6.1%)	2/13 (13.3%)	0.256 ^a^
Median follow up duration, months	65.9 (1–136)	55.6 (5.7–123.5	0.981
Mortality rate	11/203 (5.1%)	4/11 (26.7%)	0.001 ^a^

ATG: anti-thymocyte globulin, BKVAN: BK virus-associated nephropathy, BMI: body mass index, CAN: chronic allograft nephropathy, CAD: coronary artery disease, DM: diabetes mellitus, DN: diabetic nephropathy, GFR: glomerular filtration rate, GN: glomerulonephritis, HLA: human leukocyte antigen, HT: hypertension, MMF: mycophenolate mofetil, MYF: mycophenolic acid, PKD: polycystic kidney disease, TIN: tubulointerstitial nephritis. ^a^: Pearson Chi square test; ^b^: Mann Whitney-U test, ^c^: Independent sample *t* test.

**Table 2:** Donor demographic characteristics and clinical characteristics.

Parameter	No Graft Loss (n = 214)	Graft Loss Is Present (n = 15)	*p*
Donor gender, female/male (%)	135/79 (63.1%)	11/4 (73.3%)	0.425 ^a^
Donor age	43 (22–74)	47 (21–62)	0.348 ^b^
Donor BMI, kg/m^2^	26.3 (16.6–39.5)	26,8 (19.9–38)	0.609 ^b^
Pre-transplantation donor HT Yes/No (%)	13/204 (6%)	2/13 (13.3%)	0.251
Pre-transplantation donor CAD Yes/No (%)	5/212 (2.3%)	1/14 (6.7%)	0.850
Donor operation type			
Open nephrectomy	90 (42.1%)	60 (40%)	0.876 ^a^
Laparoscopic nephrectomy	124 (57.9%)	9 (60%)	
Kidney removed left/right (%left)	142/72 (%66.4)	13/2 (86.7%)	0.104 ^a^
Number of arteries	1 (1–3)	1 (1–2)	0.760 ^b^
Donor GFR, mL/min/1.73 m^2^	111.3 ± 11.5	106.3 ± 10.7	0.108 ^c^
Donor blood group			
O	112 (52.3%)	7 (46.7%)	
A	58 (27.1%)	4 (26.7%)	0.196 ^a^
B	38 (17.8%)	2 (13.3%)	
AB	6 (2.8%)	2 (13.3%)	

BMI: body mass index, CAD: coronary artery disease, GFR: glomerular filtration rate, HT: hypertension. ^a^ Pearson Chi square test; ^b^ Man Whitney-U test; ^c^ Independent sample *t* test.

**Table 3:** Correlation between the donors’ initial GFR, and both the discharge and final follow-up creatinine/GFR measurements.

		Recipients’ Post-Transplant Discharge Creatinine	Recipients’ Post-Transplant Discharge GFR	Recipients’ Final Creatinin	Recipients’ Final GFR
Donor eGFR	Correlation Coefficient (r)	−0.219 *	0.255 *	−0.240 *	0.302 *
	*p*	0.001	<0.001	<0.001	<0.001

* Spearman correlation test. eGFR: estimated glomerular filtration rate, GFR: glomerular filtration rate.

**Table 4:** Logistic regression analysis results of risk factors causing graft loss.

	Univariate Logistic Regression			Multivariate Logistic Regression		
	CI (%95)	OR	*p*	RR CI (%95)	OR	*p*
Recipient characteristic						
Gender	Referance male					
	0.100–1.331	0.365	0.127			
Age	0.921–1.013	0.966	0.149			
BMI	0.747–1.007	0.867	0.062			
Induction						
No	Referance			Referance		
Simulect	0.000	0.000	0.999	0.000	949	0.999
ATG	0.878–7.350	2.541	0.085	0.000	2087	0.999
Pre-transplatation HT	1.441–12.425	4.232	0.009	1.937–28.882	7.479	0.004
Post-transplantation HT	0.608–12.645	2.773	0.188			
CAD	0.485–11.675	2.379	0.286			
Graft biopsy	3.416–37.132	11.262	<0.001	5.387–113.609	24.74	<0.001
Donor characteristics						
Kidney side						
left	Referance					
right	0.067–1.381	0.303	0.123			
Blood group						
O	Referance			Referance		
A	0.310–3.924	1.103	0.879	0.269–5.342	1.199	0.812
B	0.168–4.230	0.842	0.835	0.060–4.638	0.527	0.564
AB	0.906–31.410	5.333	0.064	0.987–83.106	9.055	0.051
GFR	0.918–1.009	0.962	0.089	0.870–0.983	0.925	0.013

ATG: anti-thymocyte globulin, BMI: body mass index, CAD: coronary artery disease, CI: confidence interval, GFR: glomerular filtration rate, HT: hypertension, OR: odds ratio, RR: relative risk.

**Table 5:** Donor and recipient sex pairs were compared to asses clinical outcomes based on sex differences.

	Male Recipient		*p*	Female Recipient		*p*	*p* *
Recipient Characteristic	Female Donor	Male Donor		Male Donor	Female Donor		
Age	37 (18–66)	31 (18–58)	0.152	37.5 (18–69)	31 (18–59)	0.008	0.023
Recipient BMI, kg/m^2^	23.3 (15.8–34.2)	21.9 (17.7–34.6)	0.942	23 (16.4–39.8)	21 (16.2–36.7)	0.068	0.107
Induction							
No	4 (3.8%)	2 (8.6%)		2 (4.2%)	2 (4.8%)		
Simulect	23 (22.1%)	12 (34.3%)	0.15	12 (25%)	12 (28.6%)	0.91	0.666
ATG	77 (74%)	20 (57.1%)	2	34 (70.8%)	28 (67.2%)	3	
Creatinine atdischarge (mg/dL)	1.19 (0.47–6.58)	1.17 (0.68–1.62)	0.297	0.86 (0.45–2.1)	0.88 (0.46–2.2)	0.607	<0.001
GFR at discharge (mL/dk/1.73 mL^2^)	78.8 ± 28.4	86.7 ± 23.5	0.099	88.3 ± 26.7	87.9 ± 25.5	0.951	0.059
Final creatinine	1.29 (0.7–12.2)	1.2 (0.8–6.1)	0.232	0.9 (0.54–8.23)	0.95 (0.56–8.7)	0.786	<0.001
Final GFR	66 (5–118)	78 (11–128)	0.173	72.9 ± 29.9	75.9 ± 29.6	0.641	0.118
Hospitalization days	9 (6–42)	8 (6–27)	0.197	9 (6–39)	9 (6–30)	0.616	0.609
Greft biopsy, yes/no (%)	30/74 (28.8%)	7/28 (20%)	0.306	10/38 (20.8%)	6/36 (14.3%)	0.418	0.252
Biopsy result							
Normal	7 (23.3%)	2 (28.6%)					
Rejection	12 (40%)	2 (28.6%)		1 (11.1%)	1 (16.7%)		
BKVAN	3 (10%)	0 (0%)	0.777	6 (66.7%)	3 (50%)	0.937	0.935
GN	4 (13.3%)	2 (28.6%)		1 (11.1%)	1 (16.7%)		
CAN	4 (13.3%)	1 (14.3%)		1 (11.1%)	1 (16.7%)		
Biopsy result							
Normal	1 (3.3%)	0 (%0)	1.000	0 (%0)	0 (0%)	1.000	0.862
Anormal	29 (96.7%)	7 (%100)		9 (%100)	6 (100%)		
Recipient blood group							
0	24 (23.1%) ^d^	15 (%42.9) ^e^		14 (%39.2)	14 (33.3%)		
A	39 (37.5%) ^d^	13 (%37.1) ^d^	0.042	18 (%37.5)	16 (38.1%)	0.495	0.257
B	30 (28.8%) ^d^	3 (%8.6) ^e^		11 (%22.9)	11 (26.2%)		
AB	11 (%10.6) ^d^	4 (%11.4) ^d^		5 (%10.4)	1 (2.4%)		
Time spent on dialysis, months	2 (0–240)	3 (0–197)	0.622	2.5 (0–132)	0 (0–120)	0.243	0.649
Preemptive, Yes/No (%)	34/70 (%32.7)	14/21 (%40)	0.432	20/28 (%41.7)	21/21 (%50)	0.428	0.257
Number of mismatches	3.5 (0–6)	3 (0–6)	0.025	3.5 (0–6)	3 (0–5)	0.008	0.004
Primary Kidney Disease							
DN	11 (%10.6)	5 (%14.3)		3 (%6.3)	0 (%0)		
HT	16 (%15.4)	0 (%0)		12 (%25)	8 (%19)		
GN	10 (%9.6)	5 (%14.3)		3 (%6.3)	3 (%7.1)		
Obstructive pathologies	8 (%7.7)	3 (%8.6)		5 (%10.4)	4 (%9.5)		
PKD	1 (%1)	1 (%2.9)	0.091	0 (%0)	1 (%2.4)	0.639	0.250
TIN	1 (%1)	3 (%8.6)		1 (%2.1)	0 (%0)		
Amyloidosis	1 (%1)	0 (%0)		2 (%4.2)	1 (%2.4)		
Hereditary	0 (%0)	1 (%2.9)		1 (%2.1)	1 (%2.4)		
Unknown etiologies	56 (%53.8)	17 (%48.6)		21 (%43.8)	24 (%57.1)		
Pre-transplantation recipient							
HT	34/70 (%32.7)	9/26 (%74.3)	0.440	13/35 (%27.1)	9/33 (%21.4)	0.533	0.550
DM	7/97 (%6.7)	0/35 (%0)	0.115	4/44 (%8.3)	1/41 (%2.4)	0.367	0.260
CAD	9/95 (%8.7)	3/32 (%8.6)	1.000	2/46 (%4.2)	1/41 (%2.4)	1.000	0.453
Post-transplantation							
HT	79/25 (%76)	28/7 (%80)	0.623	33/15 (%68.8)	23/19 (%54.8)	0.172	0.050
DM,	29/75 (%27.9) ^d^	4/31 (%11.4) ^d,e^	0.048	13/35 (%27.1) ^d,e^	3/39 (%7.1) ^e^	0.014	0.014
CAD	9/95 (%8.7)	3/32 (%8.6)	1.000	2/46 (%4.2)	1/41 (%2.4)	1.000	0.453
Graft loss	10/94 (%9.6)	2/33 (%5.7)	0.477	2/46 (%4.2)	1/41 (%2.4)	1.000	0.351
Mortality rate	11/93 (%10.6)	1/34 (%2.9)	0.295	3/45 (%6.3)	0/42 (%0)	0.245	0.090
Donor age	42 ± 11.4	43.4 ± 11.7	0.550	44 (21–61)	46.8 (25–74)	0.128	0.180
Donor BMI, kg/m^2^	26.7 (19.6–39.5)	26.4 (19.1–34)	0.325	25.6 ± 3.6	27.6 ± 4.7	0.035	0.093
Donor operation type			0.293			0.172	0.377
Open nephrectomy	40 (%38.5)	17 (%48.6)		24 (%50)	15 (%35.7)		
Laparoscopic nephrectomy	64 (%61.5)	18 (%51.4)		24 (%50)	27 (%67.3)		
Kidney removed left/right (%left)	74/30 (%71.2)	24/11 (%68.6)	0.772	32/16 (%66.7)	25/17 (%59.5)	0.483	0.596
Number of arteries	1 (1–3)	1 (1–3)	0.864	1 (1–2)	1 (1–3)	0.783	0.590
Donor GFR, mL/min/1.73 m^2^	111.5 ± 11.8	111.7 ± 11.8	0.913	110.8 ± 11.9	109 ± 9.9	0.432	0.712
Donor GFR/Recipient BMI	4.85 ± 0.92	4.83 ± 1	0.905	4.7 (2.5–7.4)	5.5 (2.4–6.7)	0.143	0.296
Donor blood group							
0	57 (%54.8)	18 (%51.4)		24 (%50)	20 (%47.6)		
A	27 (%26)	11 (%31.4)	0.495	12 (%25)	12 (%28.6)	0.797	0.461
B	16 (%15.4)	3 (%8.6)		11 (%22.9)	10 (%23.8)		
AB	4 (%3.8)	3 (%8.6)		1 (%2.1)	0 (%0)		

ATG: anti-thymocyte globulin, BMI: body mass index, BKVAN: BK virus-associated nephropathy, CAD: coronary artery disease, CAN: chronic allograft nephropathy, DM: diabetes mellitus, DN: diabetic nephropathy, GFR: glomerular filtration rate, GN: glomerulonephritis, HT: hypertension, PKD: polycystic kidney disease, TIN: tubulointerstitial nephritis. ^d,e^: The different superscript means there is difference between the groups. * This is the *p*-value determined as a result of the statistical analysis between the four gender groups.

**Table 6:** Clinical outcomes with a donor median age of 43 years.

	Donor Age		*p*
	<43 (n = 111)	≥43 (n = 118)	
Donor GFR	118 (83–143)	107 (73–131)	<0.001 ^a^
Donor BMI	25.9 (16.6–36.8)	27.1 (18.9–39.5)	0.001 ^a^
Recipient GFR at discharge (mL/dk/1.73 mL^2^)	88.3 ± 23.4	79.4 ± 26.6	0.009 ^b^
Donor final creatinin	1.09 (0.56–9.8)	1.24 (0.54–12.2)	0.026 ^a^
Donor final GFR	75 (5–135)	65.5 (5–126)	0.008 ^a^
Median follow up time, month	78.6 (8.4–136)	59 (1–136.5)	0.029 ^a^
Graft loss, yes/no (yes %)	6/105 (%)	9/109 (%)	0.497 ^c^
Mortality rate, yes/no (yes %)	5/106 (4.5%)	10/108 (8.5%)	0.225 ^c^

BMI: body mass index, GFR: glomerular filtration rate. ^a^ Man Whitney-U test; ^b^ Independent sample *t* test; ^c^ Pearson Chi square test.

## Data Availability

The datasets used and/or analyzed during the current study are available from the corresponding author on reasonable request.
